# Supplementary material for: Causal interplay between lactose intolerance and gut microbiota: a combined bidirectional Mendelian randomization and in vivo validation study
Source: Front Nutr. 2026 Jun 1;13:1803337. doi: 10.3389/fnut.2026.1803337 (PMC13265576; doi:10.3389/fnut.2026.1803337)
Supplement: Supplementary file 1 [file Data_Sheet_1.zip › supplementary materials/Forward/leave one out cross validation/ebi-a-GCST90027566.finngen_R12_E4_LACTONAS.pdf]

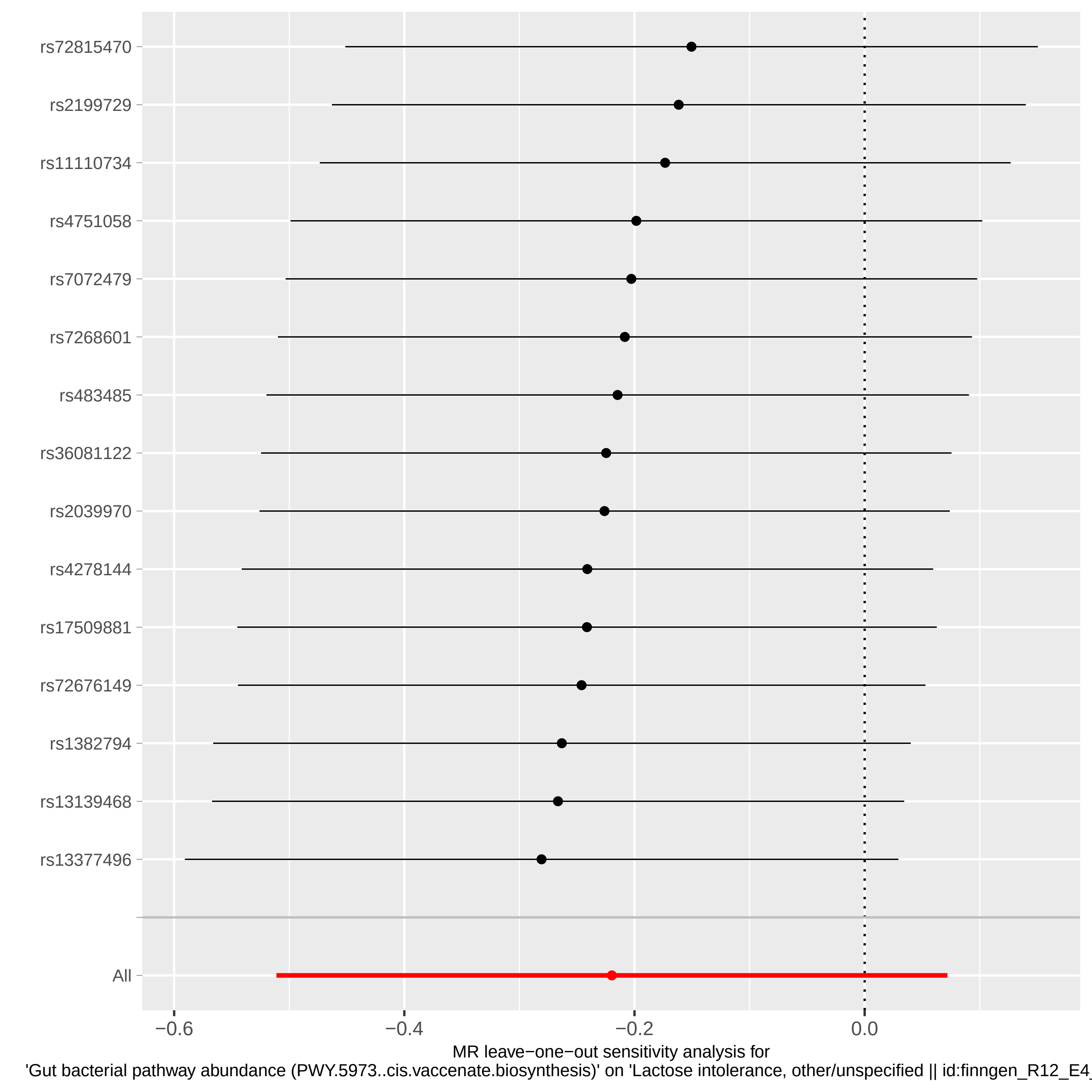
rs72815470
rs2199729
rs11110734
rs4751058
rs7072479
rs7268601
rs483485
rs36081122
rs2039970
rs4278144
rs17509881
rs72676149
rs1382794
rs13139468
rs13377496
All
−0.6
−0.4
−0.2
0.0
MR leave−one−out sensitivity analysis for
'Gut bacterial pathway abundance (PWY.5973..cis.vaccenate.biosynthesis)' on 'Lactose intolerance, other/unspecified || id:finngen_R12_E4_
